# Extreme ultra-low lasing threshold of full-polymeric fundamental microdisk printed with room-temperature atmospheric ink-jet technique

**DOI:** 10.1038/srep10623

**Published:** 2015-05-29

**Authors:** Hiroaki Yoshioka, Tomoya Ota, Cong Chen, Soichiro Ryu, Kei Yasui, Yuji Oki

**Affiliations:** 1 Graduate School and Faculty of Information Science and Electrical Engineering, Kyushu University, Fukuoka 819-0395, Japan; 2 NISSAN CHEMICAL INDUSTRIES, LTD, Chiba 274-0052, Japan

## Abstract

We experimentally demonstrated an extreme ultra-low lasing threshold from full-polymeric fundamental microdisk cavities fabricated by a novel fabrication method, the ink-jet printing method, which is much simpler and easier than previous methods such as lithography. The ink-jet printing method provides additive, room-temperature atmospheric, rapid fabrication with only two steps: (i) stacking cladding pedestal and waveguiding disk spots using the ink-jet technique, and (ii) partial etching of the cladding pedestal envelope. Two kinds of low-viscosity polymers successfully formed microdisks with high surface homogeneity, and one of the polymers doped with LDS798 dye yielded whispering-gallery-mode lasing. The fundamental disks exhibited an extremely ultra-low lasing threshold of 0.33 *μ*J/mm^2^ at a wavelength of 817.3 nm. To the best of our knowledge, this lasing threshold is the lowest threshold obtained among both organic and inorganic fundamental microdisk cavity lasers with a highly confined structure.

Many research studies on microcavities have been progressively conducted^1^,^2^,^3^,^4^,^5^,^6^,^7^,^8^,^9^,^10^,^11^,^12^,^13^,^14^,^15^,^16^,^17^,^18^,^19^,^20^ because microcavities have a variety of potential applications such as integrated optics, sensing^1^,^2^,^3^,^4^,^5^,^6^, low-threshold lasers^7^,^8^,^9^,^10^,^11^,^12^,^13^,^14^,^15^,^16^,^17^, nonlinear optics^18^, and optical signal processing^19^ owing to their high optical power storage near specific resonant frequencies, which leads to a high quality factor (Q-factor) that is supportive of whispering-gallery modes (WGMs). Microcavities with various shapes such as spheres^20^, fibre rings^21^, and disks (including toroid and conical disks)^11^,^12^,^13^,^14^,^15^,^16^,^17^,^22^,^23^ have been reported in the past years. In particular, microdisks are practical and attractive for applications requiring peculiar fabrications to flat surfaces, curved surfaces, large areas, and so on. Actually, some of attractive applications of microdisks such as on-chip microcavity nanocrystal quantum dot laser^10^ and split-mode microcavity Raman laser sensor^24^ were reported. Additionally, fabrications using organic materials are attractive for use as integrated optics owing to their easy fabrication, flexibility, and compatibility with functional organic molecules.

Many microdisks fabricated by various methods and materials have been previously reported with low-lasing thresholds or high Q-factors^11^,^12^,^13^,^14^,^15^,^16^,^17^,^22^,^23^. Focusing on low-lasing thresholds, several superior low thresholds were reported in the disk-shaped category using both organic and inorganic materials^11^,^12^,^13^,^14^,^15^,^16^,^17^. As a fundamental microdisk laser in the organic category, a pyrromethene 597-doped OrmoComp^®^ microdisk with a diameter of 47 *μ*m exhibited a lasing threshold of 13.8 *μ*J/mm^2^.^11^ As a conical microcavity laser in the organic category, a rhodamine 6G-doped polymethyl methacrylate (PMMA) microcavity with a diameter of 40 *μ*m exhibited a lasing threshold of 2.63 *μ*J/mm^2^.^12^ As a polymer microtoroid laser with a highly confined structure, tris(8-quinoline) aluminium 4-(dicyanomethylene)-2-methyl-6-(p-dimethylaminostyryl)-4H-pyran (Alq_3_:DCM)-coated PMMA microtoroid exhibited a lasing threshold of 0.88 *μ*J/mm^2^ at a diameter of 40 *μ*m^13^. As an organic/inorganic hybrid microtoroid laser, a -conjugated polymer-coated silica microtoroid with a diameter of 36 *μ*m exhibited a lasing threshold of 0.42 *μ*J/mm^2^.^14^ As inorganic microcavity lasers, Er^3+^ doped SiO_2_ microtoroid (52 *μ*m)^15^, Er^3+^ doped on-chip SiO_2_ microdisk (45 *μ*m)^16^ and Er^3+^:Yb^3+^ co-doped silica microtoroid (46 *μ*m)^17^ exhibited lasing thresholds of 22.7 *μ*J/mm^2^, 10.0 *μ*J/mm^2^ and 38.2 *μ*J/mm^2^, respectively. These thresholds are shown with excitation energy density, which is defined by dividing the incident energy by the area of the disk, conical structure, or toroid. The injection power is converted to incident energy by assuming that the gain media absorbs the energy during its fluorescence lifetime.

Although ultra-low lasing thresholds were reported in these previous studies^11^,^12^,^13^,^14^,^15^,^16^,^17^,^22^,^23^, these thresholds were almost performed by microtoroids or conical microcavities, which were formed from reflowed microdisks to increase the Q-factor. These microdisks were fabricated using a subtractive method such as the proton beam writing method^11^ or lithography with processes including (i) photolithography to create a disk, (ii) etching of the substrate, and (iii) heat reflow to improve surface homogeneities^12^,^13^,^14^,^15^,^16^,^17^,^22^,^23^. Additionally, microcavities with a strong confinement require extra treatment. These subtractive methods are suitable for mass fabrication in large areas. However, subtractive methods produce unnecessary waste materials, requires large energy consumption, and is unsuitable for on-site fabrication. Moreover, it is challenging to conduct all processes on demand or under room-temperature (RT) atmospheric conditions.

In this study, we propose an ink-jet printing method as a novel, simple fabrication process for a full-polymeric fine microdisk, which is very superior at producing low waste materials, requires little energy consumption, and is suitable for on-site fabrication because it is an additive method. The ink-jet technique is attractive for the fabrication of micro structures. Micro droplet cavities in elastomer^25^ and photonic arrays from self-organized chiral nematic liquid crystals^26^ were reported by the ink-jet technique. The fabrication procedure consists of only two steps: (i) stacking of the cladding pedestal and waveguiding disk using the ink-jet technique, and (ii) partially etching the cladding disk envelope. Rapid fabrication can be expected because these two steps can be processed in a short time. Pedestals and disks fabricated by the ink-jet technique have fine surface homogeneity because of their surface tension. Additionally, any organic dopants (chromophores, antibodies, proteins, and so on) can be introduced in the fabrication process because ink-jet printing is performed under open air and RT. Coating or doping organic dopants to organic or inorganic microdisks as reported by other groups^11^,^12^,^13^,^14^,^15^,^16^,^17^,^22^,^23^ are limited to fabricated microdisk structures because fabrications of these microdisks strongly stimulate organic dopants during the lithography and heat reflow process. We demonstrated an extreme ultra-low lasing threshold of 0.33 *μ*J/mm^2^ at a wavelength of 817.3 nm using an ink-jet-printed microcavity with the fundamental disk structure. Comb-shaped laser spectra were obtained from the LDS798 dye pre-doped microdisk. In spite of the fundamental microdisk structure cavity, this lasing threshold is lower than that of microtoroids or conical microcavities, which were formed from reflowed microdisks to increase the quality factor. To the best of our knowledge, this lasing threshold is the lowest threshold obtained among both organic and inorganic fundamental microdisk cavity lasers with a highly confined structure.

## Results

### Microdisk fabrication by the ink-jet printing method

**Figure 1:**
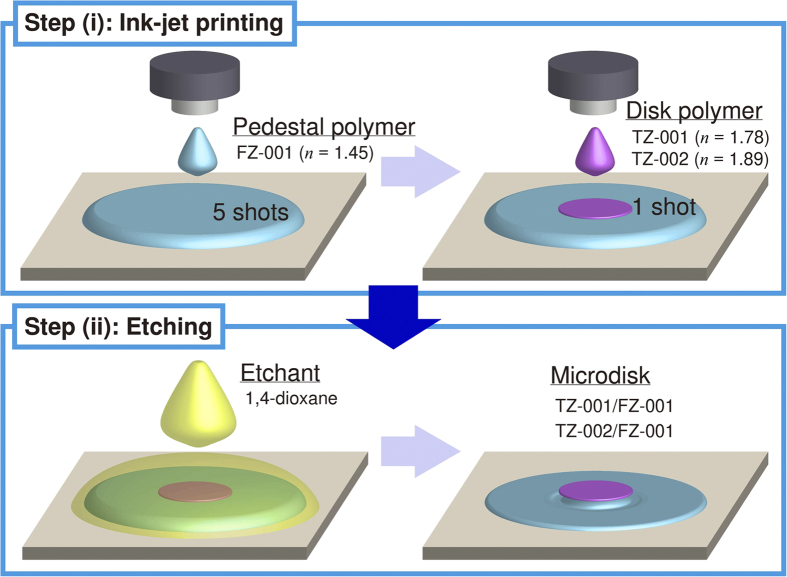
Schematic of microdisk printing by the ink-jet printing method. The fabrication procedure consists of only two steps. Step (i) is the printing of a cladding pedestal and waveguiding disk layers using the ink-jet technique. Step (ii) is the wet-etching of the cladding pedestal envelope using an organic solvent only for the cladding material.

Figure 1 shows the microdisk fabrication procedure using the ink-jet printing method. The fabrication procedure consists of two steps. First, the cladding pedestal and waveguiding disk were stacked using the ink-jet technique, where the diameter of the cladding pedestal was larger than that of the waveguiding disk. Subsequently, an etchant droplet for the cladding pedestal was dropped on the stack, and the exposed envelope of the cladding pedestal was partially etched until the etchant was evacuated. The whole process can be finished within several tens of seconds under RT atmosphere.

To satisfy the waveguiding condition, the microdisk resonator needed to be fabricated with micrometre-order thickness using a single shot of the ink-jet. Additionally, the thickness was important for maintaining the disk structure after the etching procedure. However, a general linear polymer is too viscous to eject from the ink-jet system under the required concentration. Thus, a linear polymer is not applicable for thick disk printing; it is only suitable for thin-film fabrication. In order to handle a high-concentration solution using the ink-jet technique, we considered hyperbranched polymers^27^. In this research, the novel fluorine-based hyperbranched polymer FZ-001 (*n* = 1.45) was used as the cladding pedestal and low-refractive-index layer. For the waveguiding disk, two kinds of novel triazine-based hyperbranched polymers with high refractive indices were adopted: TZ-001 (*n* = 1.78) and TZ-002 (*n* = 1.89). These hyperbranched polymers have relatively high refractive indices and high heat resistance.

**Figure 2:**
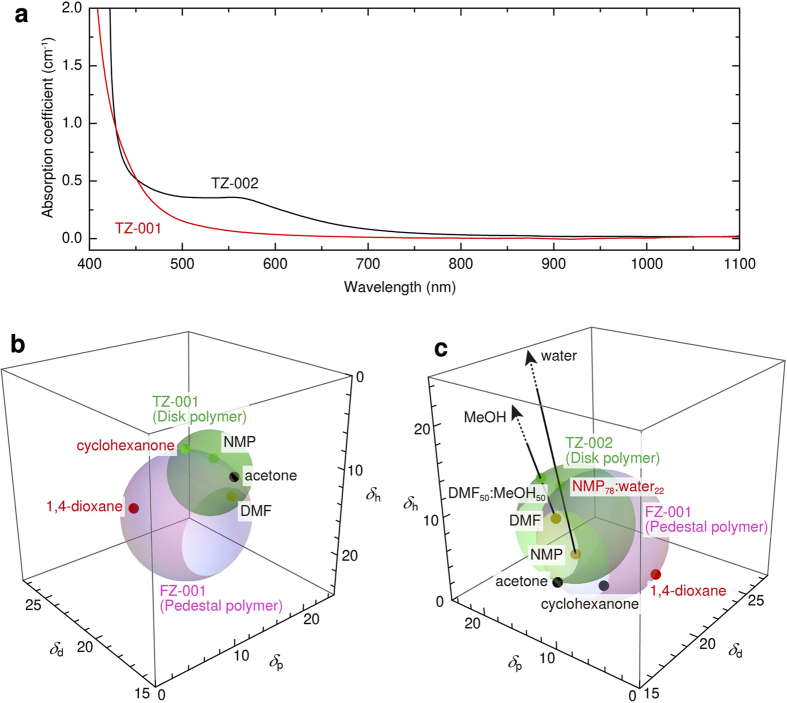
Absorption coefficient of hyperbranched polymers for the waveguiding disk and the solubility characteristics in HSP three-dimensional space. (**a**) Absorption coefficient of TZ-001 and TZ-002. (**b**) HSP spheres of TZ-001 and FZ-001. (**c**) HSP spheres of TZ-002 and FZ-001. The interior of the sphere has a solubility of 10 wt.%. The red, green, yellow, and black dots show the sphere inside of the cladding polymer (FZ-001), sphere inside of the disk polymer (TZ-001 or TZ-002), sphere inside of both polymers, and sphere outside of both polymers, respectively.

In the fabrication, two microdisks were printed using polymer pairs with ‘different’ and ‘similar’ solubility: TZ-001/FZ-001 and TZ-002/FZ-001. The TZ-001 disk was fabricated for the lasing investigation because the TZ-001 polymer has high transparency over the visible to near infrared (NIR) region, as shown in Fig. 2(a). Although the fabrication of the TZ-002 disk is challenging because its solubility is similar to that of the cladding polymer FZ-001, the fabrication of the TZ-002 disk was demonstrated using this material pair for the development of industrial applications.

First, we show step (i), which describes the stacking cladding pedestal and waveguiding disk using the ink-jet technique. When these hyperbranched polymers are used in the ink-jet technique, the combination of solvents for the two polymers is important. In particular, the solvent for waveguiding polymers must be very carefully selected because the solvent must not dissolve the cladding material FZ-001. Otherwise, the solvent for the waveguiding disk can damage the surface of the cladding pedestal. However, high-refractive-index polymers, including hyperbranched polymers, generally have lower solubility for most general solvents. The Hansen solubility parameters (HSPs)^28^ were used to satisfy the solvent requirements for disk materials. A solvent has three parameters: (1) the energy *δ*_*d*_ from dispersion forces between molecules, (2) the energy *δ*_*p*_ from the dipolar intermolecular force between molecules, and (3) the energy *δ*_*h*_ from hydrogen bonds between molecules in the HSP. Then, the distance between each solvent plot in the three-dimensional plots of these parameters exhibit the magnitude of solubility. In other words, a solvent pair with low distance between their plots shows high solubility for each other. Additionally, the HSPs for solutes have a spherical soluble range (shown as radius *R*) with the centre of plot, which consists of *δ*_*d*_, *δ*_*p*_, and *δ*_*h*_, and a solvent in the sphere can be defined such that there is a known solubility between the solvents. In particular, these four parameters can be described by the relation 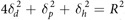. The HSP decision for hyperbranched polymers is detailed in the Methods section.

Regarding the solvent selection for a polymer pair with different solubility, first, we show the procedure for selecting the solvent for the cladding polymer FZ-001 and disk polymer TZ-001 using HSP. Figure 2(b) shows the solubility characteristics of FZ-001 (*δ*_*d*_ = 17.29, *δ*_*p*_ = 7.60, *δ*_*h*_ = 11.37, *R* = 6.3) and TZ-001 (*δ*_*d*_ = 17.64, *δ*_*p*_ = 11.21, *δ*_*h*_ = 8.20, *R* = 4.2) in the HSP three-dimensional space. Selecting a solvent in the case of a pair with different solubility is easy because many single solvents are included in the large unshared area of the HSP sphere. A single solvent of 1,4-dioxane (*δ*_*d*_ = 17.5, *δ*_*p*_ = 1.8, *δ*_*h*_ = 9.0) is suitable for the cladding polymer FZ-001 because the HSP of 1,4-dioxane is mapped on the inside of the FZ-001 sphere and the outside of the TZ-001 sphere, as shown in Fig. 2(b). The solvent 1,4-dioxane was used with a concentration of 10 wt.%. On the other hand, cyclohexanone (*δ*_*d*_ = 17.8, *δ*_*p*_ = 8.4, *δ*_*h*_ = 5.1) is a single solvent that dissolves the disk polymer TZ-001. Cyclohexanone with a concentration of 10 wt.% was used for printing the TZ-001 disk. Therefore, a laser-gain chromophore LDS798 (Exiton Corp.) was doped into the disk ink with a concentration of 5 mM for lasing.

Regarding the solvent selection for a polymer pair with similar solubility, we show the procedure for selecting a solvent for the cladding polymer FZ-001 and disk polymer TZ-002 using HSP. Figure 2(c) shows the solubility characteristics of FZ-001 (*δ*_*d*_ = 17.29, *δ*_*p*_ = 7.60, *δ*_*h*_ = 11.37, *R* = 6.3) and TZ-002 (*δ*_*d*_ = 17.13, *δ*_*p*_ = 10.71, *δ*_*h*_ = 11.90, *R* = 5.8) in the HSP three-dimensional space. As in the TZ-002/FZ-001 pair, a single solvent of 1,4-dioxane (*δ*_*d*_ = 17.5, *δ*_*p*_ = 1.8, *δ*_*h*_ = 9.0) was selected for the cladding polymer FZ-001 because the HSP of 1,4-dioxane is mapped on the inside of the FZ-001 sphere and the outside of the TZ-002 sphere, as shown in Fig. 2(c). The solvent 1,4-dioxane was used with a concentration of 10 wt.%. On the other hand, *N*,*N*-dimethylformamide (DMF, *δ*_*d*_ = 17.4, *δ*_*p*_ = 13.7, *δ*_*h*_ = 11.3) or *N*-methylpyrrolidone (NMP, *δ*_*d*_ = 18.0, *δ*_*p*_ = 12.3, *δ*_*h*_ = 7.2) are both single solvents that dissolve the disk polymer TZ-002. However, these solvents were shared between the FZ-001 and TZ-002 spheres. To define the solvent for TZ-002, methanol (MeOH, *δ*_*d*_ = 14.7, *δ*_*p*_ = 12.3, *δ*_*h*_ = 22.3) and water (*δ*_*d*_ = 15.5, *δ*_*p*_ = 16.0, *δ*_*h*_ = 42.3) were mixed into DMF and NMP, respectively. Using this mixing, the HSPs can be linearly shifted on the line between plots of the solvents. As a result, DMF_50_:MeOH_50_ (*δ*_*d*_ = 16.1, *δ*_*p*_ = 13.0, *δ*_*h*_ = 16.8) and NMP_78_:water_22_ (*δ*_*d*_ = 17.5, *δ*_*p*_ = 13.1, *δ*_*h*_ = 14.9) were defined as solvents for the disk polymer TZ-002. In this research, NMP_78_:water_22_ with a concentration of 10 wt.% was used as a solvent for TZ-002 because the solubility of NMP_78_:water_22_ for TZ-002 is higher than that of DMF_50_:MeOH_50_, limiting the solubility of TZ-002 to 2 wt.%.

After the ink preparation was completed, the cladding pedestals and waveguiding disks of TZ-001/FZ-001 and TZ-002/FZ-001 were positioned and stacked using the ink-jet printing method. A piezoelectric ink-jet system (Microjet, IJK-200s) was used in the fabrication procedure. A high-positioning-accuracy manipulation robot (Musashi Engineering, Inc., SHOTmini SL) was used for positioning the ink-jet head. The ink-jet printing process is detailed in the Methods section. The cladding polymer spot was printed with five shots to obtain a sufficiently flat area at the centre of the disk. The cladding pedestal envelope was relatively thick because of the ‘coffee ring effect’. Next, at the centre of the pedestal, one-shot printing was carried out for the waveguiding layer.

Finally, step (ii), which regards the cladding pedestal envelope etching, was manually conducted. The solvent 1,4-dioxane in Fig. 2(b) was also used as a solvent for etching the cladding polymer FZ-001. The etching was performed under observation using microscope (ECLIPSE TE2000-U, Nikon Corp.) by dropping and soaking up 1,4-dioxane. The etching process is detailed in the Methods section. The 1,4-dioxane etchant drop etched the cladding polymer FZ-001 in 1 s. For long etching procedures (∼5 s), the disk structure was broken by the movement of etchant fluid.

**Figure 3:**
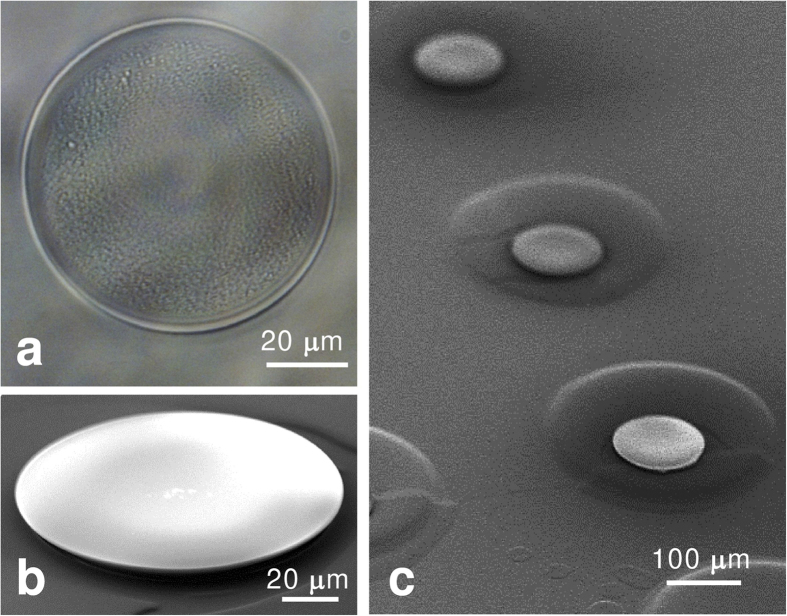
Images of printed microdisk by the RT ink-jet printing method. (**a**) Optical microscope image of a single TZ-001/FZ-001 microdisk. (**b**) SEM image of a single TZ-002/FZ-001 microdisk. (**c**) SEM image of arrayed TZ-002/FZ-001 microdisks.

Figure 3 shows an optical microscope image and a scanning electron microscopy (SEM) image of the fabricated microdisks. The TZ-001/FZ-001 microdisk with a diameter of 75 *μ*m and the TZ-002/FZ-001 microdisk with a diameter of 100 *μ*m have high surface homogeneity, as shown in Fig. 3(a), respectively. The disk height was 1 *μ*m at the thickest part according to the measurement using an atomic force microscope (VN-8000, KEYONCE Corp.). The diameter variation range performed from 75 *μ*m to 120 *μ*m when the 50-*μ*m-diameter ink-jet nozzle was used. The diameter was changed by the surface compatibility condition of ink-jet droplet on the pedestal. The low surface compatibility results in a small diameter, and sufficiently dried up surface provides the reduced diameter. Moreover, the ink-jet printing method demonstrated large-area fabrication; an example is the array structure shown in Fig. 3(c). The ink-jet printing method is suitable for the fabrication of large-area, solid structures because this method can be used for additively fabricating microdisks in a very short total time. Then ink-jet printed microdisks have enough hardness to preserve the structure due to amide bond of used hyperbranched polymers.

### Lasing characteristics of the TZ-001/FZ-001 microdisk

**Figure 4:**
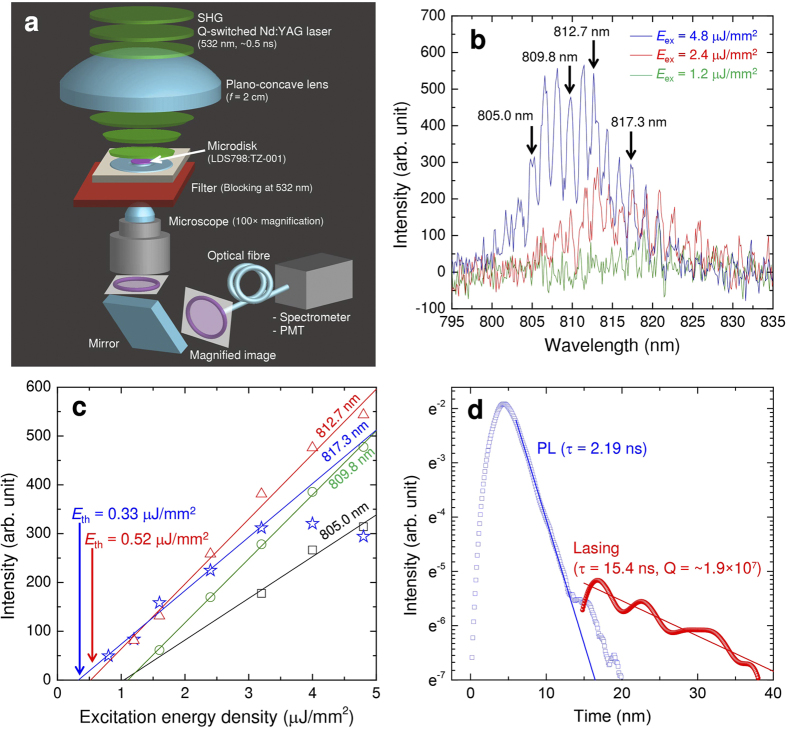
Experimental setup and lasing characteristics of LDS798:TZ-001/FZ-001 microdisk laser. (**a**) Experimental setup. (**b**) Lasing spectrum. (**c**) Input-output characteristics with a low lasing threshold. (**d**) Measured dumping curves of lasing signal and photo luminescence.

We also demonstrated a WGM laser using a microdisk. Figure 4(a) shows the experimental setup for measurement of lasing performance. In this investigation, a microdisk fabricated from the disk material TZ-001 was used because the absorption of TZ-001 between the visible and NIR range is lower than that of TZ-002. A laser-gain chromophore LDS798 was introduced to the TZ-001 microdisk with a concentration of 5 mM by pre-doping during the ink-jet printing method. The fabricated LDS798:TZ-001 microdisk sample with a diameter of 75 *μ*m was placed on the microscope for the evaluation. A passively Q-switched and frequency-doubled Nd:YAG laser (PNG-002025-040, Nanolase Corp.) was used as a pumping source. The pulse width and repetition rate were ∼0.5 ns and 100 Hz, respectively. The 532-nm pumping source was irradiated to the LDS798:TZ-001 microdisk with a maximum excitation density of 4.8 *μ*J/mm^2^. An oscillating laser was collected from the edge of the microdisk under-side by the microscope, and the WGM laser spectra were measured by an optical fibre-coupled spectrometer (Ocean Optics, HR4000) with wavelength resolution of 0.13 nm. The experimental setup is detailed in the Methods section.

First, the WGM laser spectra were evaluated as shown in Fig. 4(b). These spectra were measured with an exposure time of 30 s for the cases of the excitation density. At low excitation density, the laser oscillated at the peak wavelength of around 820 nm. When excitation density was increased, the peak wavelength was shifted to around 810 nm because of mode competition. The microdisk laser oscillated at a peak wavelength of 811.5 nm when the excitation density was 4.8 *μ*J/mm^2^. The linewidth was limited to about 1 nm due to the 0.13 nm wavelength resolution and the long exposure time (30 s) of the spectrometer. Then, a longitudinal mode width ∆*λ*_*ex*_ of 1.55 nm was obtained, and ∆*λ*_ex_ agrees with the theoretical longitudinal mode width ∆*λ*_*theory*_=*λ*^*2*^/2*rn*, where *r* is the radius of microdisk and *n* is the refractive index of the microdisk.

Next, the input–output characteristics were evaluated as shown in Fig. 4(c). These characteristics were obtained using the relative intensity of the spectra in Fig. 4(b). As a minimum lasing threshold, a lasing threshold *E*_*th*_ of 0.33 *μ*J/mm^2^ was obtained at a wavelength of 817.3 nm by determining the superlinear output onset. Comparing our work with previous reports^11^,^12^,^13^,^14^,^15^,^16^,^17^, this lasing threshold is the lowest in microdisk cavity lasers with a highly confined structure, including both organic and inorganic fundamental microdisk cavity lasers.

Finally, the lasing based cavity Q factor was evaluated by the cavity ring down method. Figure 4(d) shows dumping curves of lasing signal and photo luminescence (PL). The time of 0 s was defined at the rising start time of the pumping pulse. The dumping curve of lasing signal was illustrated without components of PL and lasing build up. These curves were measured by the experimental setup with a photomultiplier tube module (H10720-01, Hamamatsu Photonics K.K.) as shown in Fig. 4(a). The lasing signal at 0.25 mJ/mm^2^ (5 Hz) and PL were obtained from at the edge and the centre of the LDS798:TZ-001 microdisk (*φ*90 *μ*m). The decay time *τ* of 15.4 ns and 2.19 ns were obtained for lasing and PL, respectively. Computed according to *Q* *=* *ωτ*/2 with the shortest lasing wavelength of 773.6 m, the Q factor was estimated at ~1.9 × 10^7^. To be compared with Q factors of previous microdisk lasers^11^,^12^,^13^,^14^,^15^,^16^,^17^, the value of 1.9 × 10^7^ approaches the highest Q factor of the previous microdisk laser^15^. As the result, we achieved ultra-low lasing threshold.

### Numerical simulation of TZ-001/FZ-001 microdisk

**Figure 5:**
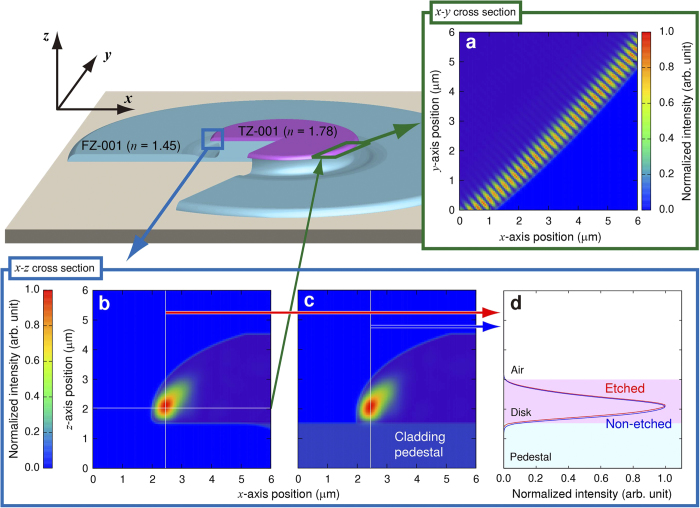
Field-intensity profiles of WGM TZ-001/FZ-001 microdisk laser modelled by three-dimensional NS-FDTD. Field-intensity profiles of the etched microdisk model at the (**a**) *x-y* cross-section and (**b**) *x-z* cross-section. (**c**) Field-intensity profile of the non-etched microdisk model at the *x-z* cross-section. (**d**) Comparison of the vertical cross-section between field-intensity profiles at the *x-z* cross-section between the etched and non-etched models.

Finally, we numerically simulated WGM lasing of the ink-jet-printed TZ-001/FZ-001 microdisk using the nonstandard finite-difference time-domain method (NS-FDTD)^29^,^30^,^31^. The simulation model is detailed in the Methods section. Figure 5 shows the result of three-dimensional NS-FDTD for the typical ink-jet-printed spot shape, which is similar to a top-flat plano-convex lens. The TZ-001/FZ-001 pair was used as a refractive-index pattern. The disk thickness and diameter were set at 3 *μ*m and 75 *μ*m, respectively. The wavelength of the simulation was set to 817.3 nm, which is the equal to the wavelength producing the ultra-low lasing threshold *E*_*th*_ of 0.33 *μ*J/mm^2^ in Fig. 4. Typical WGMs were obtained from the *x-y* cross-section at a distance of 500 nm from the disk bottom in Fig. 5(a). The evanescent wave is small because the refractive index difference of 0.78 between the core disk and cladding air is relatively high. Figure 5(b) shows the intensity profile of the *x-z* cross-section of the printed and etched microdisk model. This result revealed that the microdisk with an ink-jet-printed spot shaped like a top-flat plano-convex lens has leaky points at the bottom and side along the disk circumference. Figure 5(c) shows the intensity profile of the *x-z* cross-section of the printed and non-etched microdisk model, which shows no major difference in comparison with the profile in Fig. 5(b). However, the intensity profile of the non-etched microdisk shown in Fig. 5(c) is globally shifted to the cladding pedestal in comparison with the cross-section shown in Fig. 5(d). In other words, the non-etched microdisk fosters leaks or losses of lasing light. The etching process is important to realize high light confinement.

## Discussion

In summary, we experimentally demonstrated an extremely ultra-low lasing threshold using full-polymeric fundamental microdisk cavities fabricated by the ink-jet printing method, which is a novel fabrication method we proposed. Adopting extremely low-viscosity novel polymers enabled the first application of the ink-jet technique for thick layer stacking. Although interface roughness generally occurred because of the unavoidable co-solubility due to the limitation in solvent selectivity for the novel polymers, the rapid drying process of the ink-jet spotting could prevent interface roughness. Two novel hyperbranched polymer pairs such as TZ-001/FZ-001 and TZ-002/FZ-001 successfully formed microdisks with high surface homogeneity. Furthermore, LDS798 doping in the TZ-001/FZ-001 microdisk resulted in WGM lasing with an extremely ultra-low lasing threshold of 0.33 *μ*J/mm^2^ at a wavelength of 817.3 nm by bottom-viewing microscopic observation.The solubility between the pedestal material and the disk ink was logically controlled and optimized to incompatibility. This incompatibility occurred relatively large surface tension at the disk droplet on the pedestal. This attains the quite smooth surface on the disk-top and edge-top part. Accordingly, relatively high Q factor of ~1.9 × 10^7^ and low threshold were also performed. On the other hand, though the solvent in the disk ink can slightly dissolve the pedestal material, rapidly drying-up due to the pL amount of the droplet could avoid the degradation of the bottom interface. Thus the detectable leaked WGM output was only observed from bottom side. When the laser oscillation was observed by top-viewing microscopy, no laser spectra could be obtained; only fluorescence was observed. Therefore, the intensity profiles of the microdisk with an ink-jet-printed spot shaped like a top-flat plano-convex lens were revealed by three-dimensional NS-FDTD simulations. The numerically simulated evanescent field reveals that the laser output tended to leak from the bottom or lower surface along the disk circumference. These simulations can explain why the laser output was not observable from top-viewing microscopy but was faintly observable using bottom-viewing microscopy. To the best of our knowledge, the lasing threshold of 0.33 *μ*J/mm^2^ is the lowest threshold in microdisk cavity lasers with a highly confined structure among all organic as well as inorganic microdisk cavity lasers. Our extreme ultra-low-threshold fundamental microdisk laser can be used to further develop high-performance micro-devices such as quantum dot microcavity lasers with extremely high precision^10^ and split-mode microcavity Raman laser sensor^24^. Furthermore, the ink-jet printing method, which is simple, rapid, easy, and cost effective, opens up possibilities for future high-performance organic micro-devices.

## Methods

### The HSP decision of synthesized hyperbranched polymers

The HSP spheres of synthesized hyperbranched polymers (FZ-001, TZ-001, TZ-002) were defined by fitting a minimum sphere, which included several soluble solvents and excluded several insoluble solvents. Five soluble/insoluble solvents were considered: acetone (*δ*_*d*_ = 15.5, *δ*_*p*_ = 10.4, *δ*_*h*_ = 7), DMF (*δ*_*d*_ = 17.4, *δ*_*p*_ = 13.7, *δ*_*h*_ = 11.3), NMP (*δ*_*d*_ = 18.0, *δ*_*p*_ = 12.3, *δ*_*h*_ = 7.2), cyclohexanone (*δ*_*d*_ = 17.8, *δ*_*p*_ = 8.4, *δ*_*h*_ = 5.1), and 1,4-dioxane (*δ*_*d*_ = 17.5, *δ*_*p*_ = 1.8, *δ*_*h*_ = 9.0) with known HSPs. The soluble/insoluble decision was defined using the solubility at 10 wt.% concentration, which is the same concentration condition used for ink-jet printing. For the HSP decision of FZ-001, DMF and 1,4-dioxane were treated as soluble solvents, while acetone, NMP, and cyclohexanone were treated as insoluble solvents. For the HSP decision of TZ-001, DMF, NMP, and cyclohexanone were treated as soluble solvents, while acetone and 1,4-dioxane were treated as insoluble solvents. For the HSP decision of TZ-002, DMF and NMP were treated as soluble solvents, while acetone, cyclohexanone, and 1,4-dioxane were treated as insoluble solvents.

### Ink-jet printing

All of the ink-jet printing processes were conducted at RT. The distance between the substrate and nozzle was kept at ~1 mm during both pedestal and disk printing. Moreover, the movement speed of the ink-jet head was set at 2 mm/s to reduce the influence of air turbulence. First, a cladding polymer spot with a diameter of 250–300 *μ*m was fabricated with five printing shots using a 70-*μ*m-diameter ink-jet nozzle on a polyethylene terephthalate (PET) substrate. As ink, FZ-001-doped 1,4-dioxane was used with a concentration of 10 wt.%. The pulse voltage and pulse duration were set at 104 V and 26 *μ*s, respectively. Second, a waveguiding disk was mounted with one printing shot using a 50-*μ*m-diameter ink-jet nozzle on the central area of the cladding pedestal. When a TZ-001 disk was fabricated, TZ-001- and LDS798-dye-doped cyclohexanone ink were used. The concentration of TZ-001 and LDS798 were 10 wt.% and 5 mM, respectively. The pulse voltage and pulse duration were set at 146 V and 44 *μ*s, respectively. On the other hand, when a TZ-002 disk was fabricated, TZ-002-doped NMP_78_:water_22_ ink was used with a concentration of 10 wt.%. The pulse voltage and pulse duration were set at 120 V and 38 *μ*s, respectively.

### Etching

The etching process was manually conducted under a microscope (ECLIPSE TE2000-U, Nikon Corp.) by dropping and soaking up 1,4-dioxane that included dissolved FZ-001. When the etchant was dropped, 1,4-dioxane with a mount of 0.2 *μ*L was used for the single etching using a micro-pipet. The etchant drop of 1,4-dioxane etched the cladding polymer FZ-001 in 1 s. Then, the etchant, which included dissolved FZ-001, was soaked up from the cladding polymer on the outer side of the disk using a paper. This operation was conducted twice for one disk.

### Experimental setup for measurement of lasing performance

All investigations of lasing performance were conducted at RT. A pumping beam from the passively Q-switched and frequency-doubled Nd:YAG laser was focused onto a TZ-001 microdisk with a 300-*μ*m-diameter beam spot using a plano-concave lens with a focal length of 2 cm. A rotating variable reflective neutral density (ND) filter was used between the pumping laser and the focal lens when the excitation density changed. The intensity of the excitation density was monitored between the ND filter and the focal lens, and the excitation density was defined with consideration of the loss at the focal lens. An oscillating laser from a TZ-001 microdisk, which was set on the microscope, was collected from the edge of the microdisk under-side with 100× magnification. The WGM laser spectra were measured with an exposure time of 30 s using an optical-fibre-coupled spectrometer.

### Numerical simulation model

The numerically simulated field-intensity profiles at the TM mode (electric field parallel to the *z*-axis in Fig. 5) were obtained by calculating the wave function using the three-dimensional NS-FDTD algorithm for wave equations. All calculated field sizes were 7.9 (*x*) *μ*m × 7.9 (*y*) *μ*m × 6.5 (*z*) *μ*m. The spacing grid size *h* was set as 50 nm. At the border of the refractive index, the fuzzy boundary model was adopted for high accuracy, and the absorbing boundary condition was used.

## Additional Information

**How to cite this article**: Yoshioka, H. *et al.* Extreme ultra-low lasing threshold of full-polymeric fundamental microdisk printed with room-temperature atmospheric ink-jet technique. *Sci. Rep.* **5**, 10623; doi: 10.1038/srep10623 (2015).
